# Low back pain predict sickness absence among power plant workers

**DOI:** 10.4103/0019-5278.72241

**Published:** 2010-08

**Authors:** Ardiana Murtezani, Hajrije Hundozi, Nikola Orovcanec, Merita Berisha, Vjollca Meka

**Affiliations:** Physical Medicine and Rehabilitation Clinic, University Clinical Center of Kosovo, Kosova; 1 Department of Epidemiology and Biostatistics, Faculty of Medicine, Skopje, Macedonia; 2 National Institute of Public Health of Kosova, Medical Faculty, Kosova

**Keywords:** Low back pain, occupational, physical risk factors, sick leave

## Abstract

**Background:**

Low back pain (LBP) remains the predominant occupational health problem in most industrialized countries and low-income countries. Both work characteristics and individual factors have been identified as risk factors. More knowledge about the predictors of sickness absence from LBP in the industry will be valuable in determining strategies for prevention.

**Objectives:**

The aim of this longitudinal study was to investigate whether individual, work-related physical risk factors were involved in the occurrence of LBP sickness absence.

**Methods:**

A follow-up study was conducted among 489 workers, aged 18–65 years, at Kosovo Energetic Corporation in Kosovo. This cross-sectional study used a self-administered questionnaire to collect data on individual and work-related risk factors and the occurrence of LBP sickness absence. Logistic regression models were used to determine associations between risk factors and the occurrence of sickness absence due to LBP.

**Results:**

Individual factors did not influence sickness absence, whereas work-related physical factors showed strong associations with sickness absence. The main risk factors for sickness absence due to LBP among production workers were extreme trunk flexion (OR = 1.71, 95% CI = 1.05–2.78) as well as very extreme trunk flexion (OR = 6.04, 95% CI = 1.12–32.49) and exposure to whole-body vibration (OR = 1.75, 95% CI = 1.04–2.95).

**Conclusion:**

Reducing sickness absence from LBP among power plant workers requires focusing on the working conditions of blue-collar workers and risk factors for LBP. Increasing social support in the work environment may have effects in reducing sickness absence from LBP.

## INTRODUCTION

Low back pain (LBP) is a major health problem, not only because of the high prevalence and incidence of low back problems but also because of the important consequences for disability, the use of health services, sickness absence and early retirement.[1] Back pain also accounts for many lost working days in other countries.[2,3] LBP affects 80% of the population at some time, and is one of the most frequent reasons both for consulting a primary care physician and for taking time off work.[4,5] Symptoms associated with back disorders, in particular LBP, account for a large percentage of all sickness absence in western industrialized countries.[6,7] Sickness absence is used in occupational medicine as an important indicator of morbidity.[8,9] The rates of sickness absence and being awarded a disability pension from MSD (Musculosceletal Disorder) vary in different occupations, and tend to be higher among blue-collar workers than among white collar-workers.[8]

A few studies have shown that physical load was a risk factor for sickness absence due to LBP.[10] In the past two decades, it has been well documented that physical load caused by frequent lifting, awkward back postures and whole-body vibration is a risk factor for the occurrence of LBP. However, little is known about the impact of physical load on the long-term course of LBP and associated consequences for work disability.

The aim of this study was to analyze the relative impact of work-related factors as predictors for short- and long-term sickness absence from LBP during 1 year among power plant workers.

## MATERIALS AND METHODS

### Study design and data collection

A follow-up study was conducted among the workers, aged 18–65 years, at Kosovo Energetic Corporation in Kosovo. After written informed consent, the workers participated in a structured interview and examination survey. The workers completed a self-administered questionnaire. The questionnaire contained questions on individual data, including age, gender, height, weight, level of education, questions on sickness absence, job title and occurrence of LBP in the previous 12 months (1-year prevalence). The job titles were categorized into blue-collar workers (production workers) and white-collar workers (office workers and menagers). Work-related characteristics, such as details on years of employment and full-time work, were obtained, as well as physical load at the worksite. The questions on physical workload concerned manual materials handling, such as lifting and carrying heavy loads, static work postures, repetitive movements, awkward back postures with a bend or twisted back, prolonged sitting or standing and use of vibrating tools.

During the 1-year follow-up for all employees, data on absenteeism (occurrence, duration and diagnosis) were available from the company sickness absence register. This register, kept in the occupational health department, is mainly based on medical certifications.

### Baseline survey

After the participants completed a questionnaire they underwent a physical examination. The questionnaire included questions on age, sex, educational level and exercise behavior. The physical load factors, trunk flexion, trunk rotation and lifting of loads at work were assessed by continuous observations at the workplace. The categories of trunk flexion that were observed were defined as neutral (<30°), mild flexion (30–60°), extreme flexion (60–90°) and very extreme flexion (>90°). Assessment was performed of the number of times workers lifted a load of at least 25 kg or more than 25 kg weight. The workers have LBP defined as: pain localized in the lower back without a specific underlying cause, between the lower angle of the scapulae and above the buttocks. The physicians coded the reasons for absence according to an adapted Dutch version of the 9^th^ revision of the International Classification of Diseases (ICD-9).[2] The following diagnoses were considered to constitute sickness absence due to LBP: lumbosacral spondylosis and spondylosis of unspecified site (ICD numbers 721, 721.3, 721.42, 721.9), lumbar intervertebral disc disorders and intervertebral disc disorders and intervertebral disc disorders of unspecified site (ICD numbers 722, 722.10, 722.2, 722.52, 722.6, 722.73, 722.9), and other unspecified back disorders (ICD numbers 724, 724.2, 724.3, 724.4, 724.5, 724.9). The main measure of sickness absence used in the present study was the rate sickness absences of 7 days or longer due to LBP.

### Data on sickness absence

We used self-reported data on sickness absence. The questions about sickness absence (the dependent variables) were phrased as follows: “Have you had any sickness absence because of LBP in the past 12 months?” “How many days have you had sickness absence because of LBP in the past 12 months?” The outcome of the study was the number of days of sickness absence caused by LBP in the previous 12 months (1-year prevalence). Two indicator variables were constructed: (1) sickness absence 1–7 days because of LBP and (2) sickness absence >7 days because of LBP.

### Eligibility criteria

Inclusion criteria are LBP of at least 6 weeks duration, age from 18 to 65 years, Oswestry disability score of 25% or higher, willing and able to give informed consent. Workers are ineligible if they have been managed previously in a cognitive behavioral programme, i.e. factors associated with serious LBP pathology (including cauda equine symptoms, systemic illness [including cancer, HIV, fever], widespread neurological problems, violent trauma [fall from height, RTA, unexplained weight loss or having severe psychiatric or personality disorders]).

### Ethical approval

The study design, protocols, procedures and informed consent form were approved by the Medical Ethic Committee, Faculty of Medicine, University of Prishtina, Kosovo.

### Statistical analysis

In the statistical analysis, differences between normally distributed continuous variables were tested with the Student *t*-test and differences between categorical variables were tested with the chi-square test (x). For the continuous data, Mann–Whitney *U* test was applied. An ordinal logistic regression model were used to compute adjusted odds ratios (OR) and their 95% confidence intervals (95% CI) for the various symptoms and causes, with LBP as the dependent variable. Wald statistics was used to estimate the 95% CIs around the OR. A *P*-value of <0.05 was regarded as indicating statistical significance. Data analyses were conducted by means of the SPSS for windows 13.0 statistical package.

## RESULTS

The basic characteristics of the 489 workers with complete data are shown in Table 1. Most of the workers were blue-collar workers (87.9%), and were men (91.2%). Nine percent (*n* = 38) of the blue-collar workers and 61.1% (*n* = 36) of the white-collar workers were women. Blue-collar workers had a mean age of 48 years, with 22 years of employment, while white-collar workers had a mean age of 49 years, with 21 years of employment. More white-collar workers than blue-collar workers reported LBP during the past year (P < 0.000). Blue-collar workers were at a higher risk of long-term sickness absence as were white-collar workers at a higher risk of short-term sickness absence. During 1 year of follow-up, 20.5% (*n* = 88) of the blue-collar workers and 44.1% (*n* = 26) of the white-collar workers with sickness absence due to LBP reported a total duration of 7 days or less, 30% (*n* = 129) vs. 11.9% (*n* = 70) reported more than 7 days.

**Table 1:** Characteristics of the study population

	Group I (*n* = 430)	Group II (*n* = 59)	*P*-value
Age (years)	47.98 ± 8.97	49.44 ± 8.03	>0.236
Weight (kg)	78.13 ± 10.29	76.03 ± 10.10	>0.142
Height (cm)	174.64 ± 6.22	170.44 ± 7.68	<0.000*
Time in job (years)	21.63 ± 9.68	21.05 ± 8.32	>0.657
Educational level			
Basic	87 (20.2%)	-	<0.000*
Technical	295 (68.6%)	34 (57.6%)	
Higher	48 (11.2%)	25 (42.4%)	
Gender			
Men	392 (91.2%)	23 (38.9%)	<0.000*
Women	38 (8.80%)	36 (61.1%)	
Low back pain (*n*)	265 (61.6%)	51 (86.4%)	<0.000*
Sickness absence			
1–7 days	88 (20.5%)	26 (44.1%)	<0.000*
>7 days	129 (30.0%)	7 (11.9%)	
No sickness absence	213 (49.5%)	26 (44.1%)	

Group I, blue-collar workers; Group II, white-collar workers

* Statistically significant

Table 2 shows the effect of individual and work-related physical factors on the occurrence of short and long absences due to LBP in blue-collar workers. Absence due to low back complaints was not associated with age, weight, height, job tenure and gender. Individual factors did not exhibit a significant influence on absenteeism due to LBP among blue-collar workers. Extreme trunk flexion (OR = 1.71, 95% CI = 1.05–2.78) as well as very extreme trunk flexion (OR = 6.04, 95% CI = 1.12–32.49) and exposure to whole-body vibration (OR = 1.75, 95% CI = 1.04–2.95) were significantly associated with the occurrence of long-term sickness absence due to LBP. The work-related physical factors had a stronger relation with long absences than with short absences.

**Table 2:** Age-adjusted odds ratios (ORs) of the association between the individual, work-related physical factors and the occurrence of short-term and long-term sickness absence due to LBP in the past 12 months among the blue-collar workers (*n* = 430)

Risk factor	Short-term sickness absence (<7 days)					Long-term sickness absence (>7 days)				
	*P*#	Wald	Sig.	OR	95% CI	*P*#	Wald	Sig.	OR	95% CI
Gender	0.96					0.62				
Job title	1.00					1.00				
Age	0.67					0.008**	1.56	0.21	1.02	0.99-1.06
Weight	0.32					0.33				
Height	0.79					0.92				
Job tenure	0.21					0.009**	0.16	0.69	1.01	0.97–1.04
Trunk flexion										
Mild flexion	0.91					0.10				
Extreme flexion	0.58					0.02*	4.71	0.03*	1.71	1.05–2.78
Very extreme flexion	0.77					0.02*	4.39	0.04*	6.04	1.12–32.49
Lifting of loads										
<25 kg	0.93					0.39				
>25 kg	0.27					0.008**	2.33	0.13	1.51	0.89–2.55
Pushing or pulling heavy loads										
<1 time/h	0.79					0.33				
>1 time/h	0.99					0.28				
Heavy equipment handling	0.86					0.79				
Exposure to whole-body vibration	0.43					0.03*	4.39	0.04*	1.75	1.04–2.95
Static work postures	0.39					0.04*	1.01	0.32	0.77	0.46–1.28
Ability to change posture regularly	0.98					0.58				

*P* < 0.05*

*P* < 0.01**

*P#*-*P*-level (nonparametric significance tests - Mann-Whitney *U* test)

Table 3 presents the factors that were statistically significantly associated with the occurrence of short and long sickness absence due to LBP in white-collar workers. Ability to change posture regularly was significantly associated with the occurrence of short-term sickness absence due to LBP (OR = 3.14, 95% CI = 1.07–9.19).

**Table 3:** Age-adjusted odds ratios (ORs) of the association between the individual, work-related physical factors and the occurrence of short-term and long-term sickness absence due to LBP in the past 12 months among the white-collar workers (*n* = 59)

Risk factor	Short-term sickness absence (>7 days)					Long-term sickness absence (>7 days)				
	*P*#	Wald	Sig.	OR	95% CI	*P*#	Wald	Sig.	OR	95% CI
Gender	0.69					0.85				
Job title	1.00					1.00				
Age	0.82					0.26				
Weight	0.78					0.43				
Height	0.72					0.73				
Job tenure	0.78					0.99				
Trunk flexion/mild	0.26					0.47				
Extreme flexion	1.00					1.00				
Very extreme flexion	1.00					1.00				
Lifting of loads										
<25 kg	0.88					0.81				
>25 kg	1.00					1.00				
Pushing or pulling heavy loads										
<1 time/h	0.80					0.93				
>1 time/h	1.00					1.00				
Heavy equipments drivers	1.00					1.00				
Exposure to whole-body vibration	1.00					1.00
Static work postures	1.00					1.00				
Ability to change posture regularly	0.04*	4.34	0.04*	3.14	1.07–9.19	0.50

*P* < 0.05*

*P* < 0.01**

*P* < 0.0001***

*P#*-*P*-level (nonparametric significance tests - Mann-Whitney *U* test)

## DISCUSSION

In our study, we found that patients with LBP at the highest risk for long-term absence are blue-collar workers,[8,11] doing heavy physical work and receiving a high level of compensation.

Our study considered various factors that may influence sickness absence due to LBP. Work-related physical factors (i.e., awkward back postures, high perceived physical load, exposure to whole-body vibration) were strongly associated with the occurrence of sickness absence.The findings from the present study are in agreement with the results of three recent reviews of the literature on physical risk factors for LBP, which also showed that the evidence is strongest for trunk flexion and rotation, with manual material handling as risk factors for LBP absenteeism.[2,10,12]

There is a strong evidence for heavy work as a predictor for longer duration of sick leave.[13] Among 430 production workers included in the logistic model, sickness absence more than 7 days occurred significantly more often among workers reporting manual lifting of weights >25 kg, extreme trunk flexion, very extreme trunk flexion, exposure to whole-body vibration and static work postures (*P* < 0.01). The risk factors for sickness absence due to LBP found in this study are already known – uncomfortable working positions,[14] exposure to whole-body vibration and ability to change posture regularly.[15–17]

Hoogendoorn *et al*. and Ariens *et al*.[12] reported that work-related physical factors were more strongly associated with sickness absence than were the occurrence of LBP and neck pain.

Sitting has been associated with the risk of developing LBP. Sitting alone was not associated with the risk of developing LBP. One study reported[13] sitting and walking as not predicting prolonged work absenteeism.

Our study showed that exposure to whole-body vibration was significantly associated with the occurrence of long-term sickness absence due to LBP (OR = 1.75, 95% CI = 1.04–2.95). There is insufficient evidence for vibration as a predictive factor because only one study[13] found a significant effect of this factor for duration on sick leave. Driving a vehicle might be correlated with this factor, but was only a predictive factor if driving took more than 75% of the working day. Lis *et al*.[18] reported that the risk effect of prolonged sitting increased significantly when the factors of whole-body vibration and awkward postures were combined.

Individual characteristics such as age, height, weight and duration of employment were not predictive for low back complaints leading to absence from work.[8,9,19] Individual factors did not exhibit a significant influence on absenteeism due to LBP in the whole study population. This is not in agreement with the findings of other researchers.[12,14,20]

Non-specific LBP is an uncomfortable medical condition that causes frequent disability and absence from work.[18] Most episodes of LBP resolve fairly quickly and cause only short periods of absence from work. However, some workers with LBP miss work for several days to weeks and are at risk for more permanent disability.[21] To prevent costs and personal suffering from long-term sick leave and disability, we need to assess prognostic factors that can be identifies, which high-risk patients should focus on.[13]

In this study, physical load was assessed by detailed observations at the workplace among a random sample of workers within each occupational group. The most common observed risk factors the workers encountered were awkward working posture, manual material handling of heavy loads, working in a standing position for a long time and lack of rest. Regarding this, the following corrective measures were recommended for reducing exposure level and, consequently, preventing sickness absence in the Kosovo Energetic Corporation:

- Reducing the weight of loads that are handled manually
- Using mechanical devices such as lifter to lift and carry loads
- Designing sitting-standing workstations on the coal transportation strip
- Devising an appropriate work-rest cycle

Another possible explanation is the effect of such social factors as education and income, which have been found to predict sickness absence among industrial workers,[8] but were not considered in this study.

A particular strength of this study was that all subjects worked in the same company and were comparable for several factors such as cultural and socioeconomic factors. Disadvantage of this study is that psychological factors were not addressed and thus their potential influence on absenteeism could not be established.[9]

## CONCLUSION

Blue-collar workers were at a higher risk of sickness absence from LBP. Extreme and very extreme trunk flexion and whole-body vibration were identified as predictors for a longer duration of sick leave. A history of LBP, age, job tenure, weight and gender were of minor importance in predicting sickness absence from LBP in this industrial population. Nevertheless, we suggest that the most important strategy seems to be preventing sickness absence by reducing all the known risk factors for LBP. The working conditions of blue-collar workers, including physical risk factors,[11] had to be given special attention and had to be investigated further.
